# Self-reported hearing difficulty and its association with general, cognitive, and psychosocial health in the state of Arizona, 2015

**DOI:** 10.1186/s12889-019-7175-5

**Published:** 2019-07-04

**Authors:** Nicole Marrone, Maia Ingram, Kristi Bischoff, Emily Burgen, Scott C. Carvajal, Melanie L. Bell

**Affiliations:** 1 Department of Speech Language and Hearing Sciences, University of Arizona, 1401 E. University Blvd, Tucson, AZ USA; 2 Health Promotion Sciences, College of Public Health, University of Arizona, 1295 N. Martin, Tucson, Arizona USA; 3 Epidemiology and Biostatistics, College of Public Health, University of Arizona, 1295 N. Martin, Tucson, AZ USA

**Keywords:** Aging, Deafness/hearing loss, Cognitive functioning, Dementia, BRFSS

## Abstract

**Background:**

Hearing loss is among the leading causes of disability in persons 65 years and older worldwide and is known to have an impact on quality of life as well as social, cognitive, and physical functioning. Our objective was to assess statewide prevalence of self-reported hearing ability in Arizona adults and its association with general health, cognitive decline, diabetes and poor psychosocial health.

**Methods:**

A self-report question on hearing was added to the 2015 Behavioral Risk Factor Surveillance System (BRFSS), a telephone-based survey among community-dwelling adults aged > 18 years (*n* = 6462). Logistic and linear regression were used to estimate the associations between self-reported hearing loss and health outcomes.

**Results:**

Approximately 1 in 4 adults reported trouble hearing (23.2, 95% confidence interval: 21.8, 24.5%), with responses ranging from “a little trouble hearing” to being “deaf.” Adults reporting any trouble hearing were at nearly four times higher odds of reporting increased confusion and memory loss (OR 3.92, 95% CI: 2.94, 5.24) and decreased odds of reporting good general health (OR = 0.50, 95% CI: 0.40, 0.64) as compared to participants reporting no hearing difficulty. Those reporting any trouble hearing also reported an average 2.5 more days of poor psychosocial health per month (β = 2.52, 95% CI: 1.64, 3.41). After adjusting for sex, age, questionnaire language, race/ethnicity, and income category the association between diabetes and hearing loss was no longer significant.

**Conclusions:**

Self-reported hearing difficulty was associated with report of increased confusion and memory loss and poorer general and psychosocial health among Arizona adults. These findings support the feasibility and utility of assessing self-reported hearing ability on the BRFSS. Results highlight the need for greater inclusion of the full range of hearing disability in the planning process for public health surveillance, programs, and services at state and local levels.

## Background

Hearing loss is among the leading causes of disability in persons 65 years and older worldwide [1]. Projections of hearing loss are climbing rapidly with the aging of the population, from an estimated 44.1 million American adults in 2020 to 73.1 million American adults in 2060 [2]. Hearing health is known to have an impact on quality of life as well as social, cognitive, and physical functioning [3]. A recent consensus report from the National Academies of Sciences, Engineering, and Medicine recommended that public health agencies in the United States give priority to gaining a comprehensive understanding of the extent and impact of hearing loss in the states and other populations [3]. States need this information to set program priorities, allocate resources, and determine policies. However, efforts to develop state-level public health surveillance for hearing health and/or health outcomes among adults who are d/Deaf or hard-of-hearing have been limited so far [4–7].

Beginning in 2016, the Centers for Disease Control and Prevention (CDC) added a question on severe hearing loss or deafness to the core module of the Behavioral Risk Factor Surveillance System (BRFSS) in 2016 [8]. In Arizona, the weighted prevalence of deafness or serious difficulty hearing for adults was 7.5%. However, the survey question limits estimates to a small fraction of the hard-of-hearing population, which includes many more individuals with mild to moderate degrees of impairment [2, 9, 10]. Acquired hearing loss of *any* severity has been associated with dementia, depression, diabetes, the risk of falls, and a number of other health concerns related to healthy aging [3].

The purpose of this study was to use the BRFSS to determine the prevalence of *all* degrees of self-reported hearing loss in Arizona in 2015 and to investigate the associations between any trouble hearing with four self-reported health outcomes: general health, diabetes, cognitive functioning, and mental/psychosocial health. We hypothesized that hearing loss would be associated with each of these outcomes based on the current evidence [3, 11–14].

## Methods

The first aim of the study was to estimate the prevalence of self-reported hearing loss in Arizona. The second aim was to examine the hypothesized associations between self-reported hearing loss and self-reported health outcomes: general health, cognitive functioning, and mental/psychosocial health. The BRFSS is a cell phone/landline conducted survey that collects state-based data in collaboration with the CDC on self-reported individual health status, risk-related behaviors, and prevention activities [8]. Core modules of the survey include questions asked in all 50 states, the District of Columbia, Puerto Rico, and the U.S. Virgin Islands and each state can add supplemental modules or questions through an election process. The University of Arizona Institutional Review Board waived human subjects review for this study because BRFSS data are in the public domain.

### Accommodations for persons with hearing loss

In addition to adding a question about hearing loss, the Arizona Department of Health Services, responsible for administering the survey, also reviewed the standard BRFSS survey procedures for inclusive participation and equal access by people with hearing loss. This review led to accommodations developed in partnership with the University of Arizona and the Arizona Commission for the Deaf and the Hard of Hearing. Accommodations included: a) announcement of the availability of accommodations for full participation by persons with hearing loss in the pre-notification letter routinely sent to all randomly selected survey participants; b) a dedicated phone number to contact the state BRFSS coordinator to schedule an interview time and facilitate accommodations as requested; and c) approval from the CDC to allow those with a hearing impairment to use another household member or an American Sign Language interpreter to provide their answers to the survey. In addition, the Arizona Commission for the Deaf and Hard of Hearing prepared a captioned video in American Sign Language to introduce the BRFSS survey and communicate instructions for participation, which was made available on the BRFSS website.

### Participants

Participants included respondents aged 18 to 99 years who answered the state-added hearing question and self-reported their age (excluding responses “I don’t know” and refusal to answer) from the 2015 Arizona BRFSS (*n* = 6462).

### Self-reported hearing status

Self-reported hearing status was measured by the same question used by the National Health and Nutrition Examination Survey and the National Health Interview Survey: “Which statement best describes your hearing (without a hearing aid or other listening device)? Excellent, good, a little trouble, moderate trouble, a lot of trouble, or deaf?” We categorized self-reported hearing status as a binary variable with responses of ‘excellent’ and ‘good’ categorized as “no hearing loss” and ‘a little trouble,’ ‘moderate trouble,’ ‘a lot of trouble,’ and ‘deaf’ as “hearing loss,” following the definition used in other previous studies of self-reported hearing loss [15, 16].

### Outcome variables

The outcome variables were selected based on the rationale that hearing health is known to have impact on quality of life as well as associations with social, cognitive, and physical functioning.^3^ Conversely, we included diabetes as a variable given the evidence that it may be a risk factor for hearing loss [17, 18]. Outcome variables included general health, cognitive functioning as measured by increased confusion and memory loss, diabetes and psychosocial health. General health was treated as binary and was measured by the question, “Would you say that in general that your health is ….” We categorized the responses: “excellent”, “very good”, “good”, and “fair” as acceptable and “poor” as poor general health. Cognitive functioning was measured by the question, “During the past 12 months, have you experienced confusion or memory loss that is happening more often or is getting worse?” and was only asked to participants 45 years or older. We categorized cognitive health as binary (poor or acceptable) by a “yes” or “no” response. Diabetes was also a binary outcome and was measured by the question, “Has a doctor, nurse, or other health professional ever told you that you have diabetes?” We categorized the responses: “yes” and “yes, gestational only” as diabetic and “no,” “pre-diabetes” or “borderline diabetes” as non-diabetic. Psychosocial health remained continuous and was measured by the question, “Now thinking about your mental health, which includes stress, depression, and problems with emotions, for how many days in the past 30 days was your mental health not good?”

### Covariates

Our covariates included age, sex, race/ethnicity, household income and the respondent’s primary language (English or Spanish) measured by the language used to conduct the questionnaire. We used the imputed race variable provided by the 2015 data and collapsed the six categories into: 1) White, non-Hispanic, 2) Black, non-Hispanic, 3) Hispanic, and 4) Other race, non-Hispanic.^1^ Income was categorized in increments of $5000, from < $10,000 to >$75,000.

### Statistical analyses

All analyses were performed in SAS version 9.4 (Cary, North Carolina). The survey sampling design was accounted for in all analyses by using the survey, cluster and strata weights provided in the dataset, in SAS’s survey procedures (age, race and ethnicity, gender, geographic region, and other known characteristics of a population). Survey weighted descriptive statistics were calculated. The prevalence of self-reported hearing loss in Arizona was estimated using a proportion and 95% confidence interval. We used logistic regression to test the association of hearing with general health status, diabetes, and cognitive functioning. Linear regression was used to model the number of days of poor psychosocial health. We fit unadjusted and adjusted models for the four outcomes. Adjusted models were pre-specified and included sex, income level, age, race/ethnicity, and questionnaire language. We also performed sensitivity analyses to address missing income data, which was missing 20% of its values. In the sensitivity analysis, we used education level as a proxy for socio-economic status in each of the adjusted models. Education was missing only 0.4% of its values, and was categorized as: “less than high school,” “high school graduate,” “some college” and “college graduate.”

## Results

A total of 7946 participants responded to the 2015 Arizona BRFSS and of these, 6462 participants answered the state-added question about hearing status and were included in our sample for measuring the prevalence of self-reported hearing loss in the state of Arizona.

### Self-reported hearing loss

Of the 6462 participants with complete data for the state-added hearing question on the 2015 Arizona BRFSS, 1919 reported some hearing loss. The estimated prevalence, accounting for the sampling weights, was 23.2% (95% CI: 21.8, 24.5%). The lower confidence limit is well above the US prevalence of 16% based on responses to this question on the NHIS [19], indicating a statistically higher rate. The prevalence of mild or moderate trouble hearing (13.2 and 7.5, respectively) was higher than prevalence of a lot of trouble or deafness (2.2 and .3, respectively). Persons with self-reported hearing loss were more likely to be male (prevalence = 25.8% compared to 20.7% female and older (prevalence was < 11% for persons 44 years and younger, 22.2% for persons 45–54, 30.4% for persons 55–64 and 43.9% for persons 65 and older). The highest prevalence of self-reported hearing loss was among non-Hispanic Whites, at 28.3%, followed by the “Other race, non-Hispanic” category, at 26.0%. All other race/ethnicity groups had rates less than 17%, with Hispanics at the lowest prevalence (13.8%) (Table 1).

**Table 1:** Participant characteristics and prevalence from the 2015 Arizona Behavioral Risk Factor Surveillance System (*n* = 6462) survey. Values shown are n (%), median (interquartile range), or prevalence (95% confidence interval (CI)), and are weighted for the sampling design^a^

Variable^b^	Total (n = 6462)	Any hearing trouble (*n* = 1919)	Prevalence^d^ for total respondents (95% CI)
Any hearing trouble^c^, n (%)	1919 (23.1)		23.1 (21.8, 24.5)
Hearing, n (%)			
Excellent	2053 (36.1)	0	36.1 (34.3, 38.0)
Good	2490 (40.7)	0	40.7 (38.9, 42.6)
A little trouble	1042 (13.2)	1042 (57.0)	13.2 (2.1, 14.3)
Moderate hearing trouble	635 (7.5)	635 (32.4)	7.5 (6.7, 8.3)
A lot of trouble	219 (2.2)	219 (9.5)	2.2 (1.7, 2.6)
Deaf	23 (0.3)	23 (1.2)	0.3 (0.13, 0.41)
Age, median (inter-quartile range)	45.9 (31.1, 61.7)	60.9 (48.4, 73.2)	
Age category, n (%)			Prevalence for any hearing trouble (95%)
18–24 years	203 (11.8)	23 (5.1)	10.2 (5.6, 14.8)
25–34	405 (16.6)	45 (7.6)	10.6 (7.2, 14.0)
35–44	679 (16.6)	81 (7.8)	11.0 (8.1, 13.8)
45–54	886 (16.1)	187 (15.4)	22.2 (18.7, 25.8)
55–64	1273 (16.2)	361 (21.2)	30.4 (27.3, 33.6)
65 and older	2896 (22.7)	1193 (42.8)	43.9 (41.5, 46.2)
Sex, n (%)			
Male	2544 (47.3)	887 (52.9)	25.8 (23.7, 28.0)
Female	3918 (52.7)	1032 (47.1)	20.7 (19.0, 22.4)
Race/Ethnicity, n (%)			
Hispanic	992 (26.7)	185 (15.9)	13.8 (11.2, 16.3)
White, Non-Hispanic	4918 (62.1)	1612 (76.1)	28.3 (26.6, 30.1)
Black, Non-Hispanic	161 (3.8)	29 (2.3)	14.1 (7.0, 21.0)
Asian, Non-Hispanic	100 (2.5)	15 (1.5)	14.0 (4.1, 24.1)
American Indian/Alaska Native, Non-Hispanic	112 (3.3)	24 (2.4)	16.7 (8.4, 25.0)
Other race, Non-Hispanic	179 (1.7)	54 (1.9)	26.0 (16.9, 35.2)
Primary Language, n (%)			
English	6138 (90.3)	1862 (94.3)	24.2 (22.7, 25.6)
Spanish	324 (9.7)	57 (5.7)	13.5 (9.1, 17.8)
Education			
Less than high school	449 (14.3)	141 (14.0)	22.7 (18.0, 27.3)
High school graduate	1450 (25.1)	480 (27.2)	25.1 (22.2, 28.0)
Some college	1971 (36.4)	609 (38.6)	24.5 (22.1, 27.0)
College graduate	2565 (24.2)	679 (20.2)	19.2 (17.5, 24.0)
Income, n (%)			
<$10,000	201 (4.3)	68 (5.0)	25.9 (18.3, 33.4)
$10,000–$15,000	289 (6.1)	99 (6.7)	24.7 (18.3, 31.0)
$15,000–$20,000	389 (9.6)	107 (8.5)	19.7 (14.4, 25.1)
$20,000–$25,000	523 (11.6)	174 (12.3)	23.7 (19.0, 28.4)
$25,000–$35,000	558 (10.2)	184 (12.9)	28.0 (22.6, 33.5)
$35,000–$50,000	800 (15.4)	260 (16.8)	24.3 (20.4, 28.3)
$50,000–$75,000	819 (14.8)	234 (13.8)	20.8 (17.4, 24.2)
>$75,0000	1553 (28.0)	370 (24.1)	19.3 (16.7, 21.8)
Cognitive Functioning (Increased confusion or memory loss)^e^, n (%)			
Yes	640 (13.1)	375 (23.3)	59.0 (53.7, 64.3)
No	4468 (85.6)	1357 (74.4)	28.8 (27.1, 30.5)
Psychosocial Health^f^ (Number of days mental health not good), (median, inter-quartile range)	5.0 (2.0, 18.0)	7.8 (2.5, 24.6)	
Diabetes^g^ n (%)			
Yes	921 (10.5)	381 (17.1)	37.6 (33.4, 41.9)
Yes, gestational only	70 (1.2)	21 (1.1)	22.8 (9.1, 36.5)
No (Pre-diabetes or Borderline Diabetes)	120 (1.9)	52 (3.7)	20.8 (19.4, 22.3)
No	5342 (86.2)	1461 (77.5)	45.9 (32.6, 59.1)
General Health^h^ n (%)			
Excellent	1166 (19.5)	225 (12.3)	14.6 (12.0, 17.2)
Very Good	2080 (30.6)	525 (23.6)	17.8 (15.7, 19.9)
Good	1963 (30.6)	638 (34.7)	26.2 (23.5, 29.0)
Fair	903 (14.5)	358 (20.2)	32.3 (28.2, 36.4)
Poor	339 (4.6)	166 (8.8)	43.8 (36.5, 51.1)

^a^Due to rounding, some percentages do not add to exactly 100%

^b^Missing data rates < 2% for each variable except for income (20%)

^c^Defined as self reporting “A little trouble”, “Moderate hearing trouble”, “A lot of trouble”, or “Deaf” in response to “Which statement best describes your hearing (without a hearing aid or other listening device)?”

^d^The denominator for the variable “hearing” is the total (n = 6462). The denominators for all other prevalence estimates are the row totals for each stratum (2nd column)

^e^Self-report in response to: “During the past 12 months, have you experienced confusion or memory loss that is happening more often or is getting worse?”; asked only to participants 45 years or older

^f^Self-report in response to “Now thinking about your mental health, which includes stress, depression, and problems with emotions, for how many days in the past 30 days was your mental health not good?”

^g^ Self-report in response to: “Has a doctor, nurse or other professional ever told you that you have diabetes?”

^h^ Self-report in response to: “Would you say that in general that your health is …”

### Self-reported hearing loss and health outcomes

Results from the regression models are shown in Table 2. Unadjusted and adjusted estimates were generally similar, so we report only the adjusted estimates in the text. Adults reporting any trouble hearing were at nearly four times higher odds of reporting concerns with cognitive functioning (OR 3.92, 95% CI: 2.94, 5.24) and decreased odds of reporting good general health (OR = 0.50, 95% CI: 0.40, 0.64) as compared to participants reporting no hearing difficulty. For those with hearing loss, there was a statistically significant increase of 2.5 days of poor mental health per month (β = 2.52, 95% CI = 1.63, 3.41). Associations between hearing loss and diabetes, while statistically significant in unadjusted models, were not significant in the adjusted model (OR = 1.19, 95% CI: 0.92, 1.55). Results from the sensitivity analysis largely agreed with the primary analyses in terms of statistical significance, with the exception of diabetes, where the effect of hearing loss was statistically significant, as in the unadjusted model. The OR of 1.34 (95% CI: 1.08, 1.69) was between the adjusted OR (1.19) and the unadjusted OR (1.89), and may be due to the exclusion of participants with missing income data from the adjusted model. Sensitivity estimates for cognitive functioning were similar to unadjusted and adjusted estimates; odds of reporting good general and poor mental health days were similar to unadjusted and adjusted estimates. Neither age (dichotomized at 60 years) nor gender modified the effect of hearing category for the regression models.

**Table 2:** Association of self-reported hearing loss and cognitive functioning, diabetes, general health and number of poor mental health days

Subjective Outcome^a^	Unadjusted OR (95% CI)^b^	*p*-value	Adjusted^c^ OR (95% CI)^b^	*p*-value	Sensitivity OR (95% CI)^b,d^	*p*-value
Cognitive functioning	3.64 (2.87, 4.60)	<0.0001	3.92 (2.94, 5.24)	< 0.0001	3.76 (2.91, 4.83)	<0.0001
Diabetes	2.09 (1.71, 2.35)	<0.0001	1.19 (0.92, 1.55)	0.19	1.34 (1.08, 1.69)	0.009
Good general health	0.47 (0.39, 0.56)	<0.0001	0.50 (0.40, 0.64)	< 0.0001	0.48 (0.39, 0.58)	<0.0001
	Unadjusted regression coefficient β (95% CI)^b^		Adjusted regression coefficient β (95% CI)^b^		Sensitivity regression coefficient β (95% CI) ^b,d^	
Poor mental health days	3.43 (3.08, 3.78)	<0.0001	2.52 (1.63, 3.41)	< 0.0001	2.68 (1.87, 3.50)	<0.0001

^a^ Unadjusted models: *n* = 4989 (cognitive functioning); *n* = 6453 (diabetes); *n* = 6451 (general health); *n* = 6346 (mental health);

Adjusted models: *n* = 3952 (cognitive functioning); n = 5073 (diabetes); *n* = 5073 (general health); *n* = 5020 (mental health)

^b^ OR = Odds ratio, CI = Confidence Interval, β = Regression coefficient

^**c**^ Adjusted for sex, age, questionnaire language, race/ethnicity and income category

^d^ Adjusted for sex, age, questionnaire language, race/ethnicity and education

## Discussion

This study represents the first effort to gain state-specific population-based information in Arizona about hearing loss and associations with health outcomes. There is limited data from other states with which to compare. A published study from Michigan estimated the prevalence of self-reported hearing loss as 19% using a state-added BRFSS question in 2003: Do you now have deafness of trouble hearing in one or both ears? [6] Starting in 2016, the CDC added a question on deafness or serious difficulty hearing to the core modules of the BRFSS. Li et al. (2017) provided the first analysis of prevalence data for this question across all 50 states [7]. In this study, weighted prevalence of self-reported deafness or serious difficulty hearing was 5.8% of the adult population nationally and 7.5% in Arizona. Our study in Arizona is the first to include self-reported mild to moderate hearing difficulty, which represented the majority of people who reported hearing loss of any severity. The weighted prevalence of serious trouble hearing or deafness was lower (2.5%), yet the overall estimate of hearing trouble in the state was higher in accounting for mild or moderate trouble hearing (23.7%). Hispanics in our study were less likely than non-Hispanics to self-report hearing loss, which likely reflects a lower rate of self-report among Hispanics, also documented by Kamil et al. (2015) in their study that compared self-report to audiometric testing using the same NHIS/NHANES question [15]. We interpret this result with caution because a previous study of measured hearing ability using pure-tone audiometry among Hispanics demonstrated the level of hearing loss as comparable to non-Hispanics [2, 18].

Our findings confirmed that increased self-reported hearing trouble is associated with the hypothesized subjective health outcomes: poorer general health, change in cognitive functioning, and psychosocial health, and possibly diabetes. Adults reporting trouble hearing, even of a mild degree, were at lower odds of reporting good general health, higher odds of reporting confusion or memory loss, worse psychosocial health and possibly greater prevalence of self-reported diabetes. Our findings are supported by other cohort and longitudinal studies documenting independent associations between hearing loss and cognition, [11, 12] as well as hearing loss and psychosocial health [3, 20]. Given that the Lancet Commission on Dementia Prevention, Intervention, and Care has recently identified hearing loss as an important modifiable risk factor [14] and prevention of dementia is a public health priority [13], our findings underscore the need for further research on how to mitigate associations between hearing loss and cognitive health concerns. Research on strategies to intervene on hearing loss is particularly critical in light of the challenges that d/Deaf and hard-of-hearing adults encounter in receiving appropriate and effective health care communication [21, 22]. We hypothesized that self-reported hearing loss would be associated with diabetes based on previous findings; our results were somewhat equivocal, as the unadjusted and sensitivity models showed a statistically significant effect, but the adjusted model did not. Further research is needed to study potential associations between hearing status and other health conditions.

### Implications for policy

Our findings demonstrate the importance of state level efforts to document self-reported levels of hearing loss by severity for two reasons. First, the inclusion of a question addressing mild to moderate hearing loss captured a higher prevalence of hearing loss in Arizona than in national estimates of serious trouble hearing or deafness. Second, in our findings, any trouble hearing was associated with increased risk for negative health outcomes. Taken together, these findings encourage ongoing monitoring of disability to assist public health programs in prioritizing hearing loss as a risk factor. The data support increased attention to all degrees of hearing loss within initiatives for healthy aging for older adults and effective communication access in healthcare settings.

The inclusion of a core question in the BRFSS with response categories for mild to moderate hearing impairment would enable states to identify functional disability and consider the allocation of appropriate resources. State governments and agencies of the Deaf and the hard-of-hearing advocate for and implement policies that impact distribution of resources related to health care, education, and telecommunications. Analyses of current public health agency resources and spending generally have demonstrated that priorities vary by region [23]. Approximately 38 of the 50 states have state agencies of the Deaf and the hard of hearing [24]. Data on state-level prevalence of hearing loss and associated health concerns could therefore be important for policy and public health initiatives. A lack of data sources including persons with hearing loss makes it otherwise impossible to consider how being deaf or hard-of-hearing may impact access to care and health outcomes at a state level.

These issues are particularly important with regard to access to hearing health care, also variable across the states. Access to hearing health care and hearing aids for individuals eligible for Medicaid varies significantly from state to state [25]. Arizona, for example, offers Medicaid (AHCCCS) to adults with dependent children and childless adults up to 133% of the federal poverty level as well as adults who qualify for supplemental security income (SSI). However, AHCCCS does not provide coverage for hearing aids or rehabilitative services to adults. Among the 13 Western states, nine cover hearing aids for some adults, but only five offer Medicaid benefits to non-disabled, childless adults who are Medicaid-eligible [25]. Vocational rehabilitation, a public program funded by the Rehabilitation Services Administration is also implemented through Federal/State partnerships.

Equitable communication access in education, public, and government settings is protected under the Americans with Disabilities Act of 1990 and Section 504 of the Rehabilitation Act of 1973. The BRFSS is essential in complying with the act given that a lack of data sources including persons with hearing loss makes it otherwise impossible to consider how being Deaf or hard of hearing may impact access to care and health outcomes at a state level. We found that it was feasible to engage state public health authorities to increase accommodations for people with hearing loss to access to a state-level public health survey. These survey data were collected over a single year, but the outcomes demonstrate feasibility and success of providing accommodations to access the survey for the implementation of the question and process in future years. Subsequent efforts to further increase accessibility in future years could include translation of survey items into American Sign Language such as has been conducted in the Deaf Health Survey or the Health Information National Trends Survey in American Sign Language or through follow-up study based on an individual’s responses related to the hearing question [5].

## Limitations

Some limitations of our study are inherent to challenges of the BRFSS as a statewide population study. The BRFSS is a cross sectional study, and therefore cannot establish causality across variables. The BRFSS protocol randomly selects individuals with personal cell phones and/or landlines, and therefore does not capture individuals living in nursing homes or other institutions who may be significantly impacted by hearing loss. Another potential limitation is the use of a telephone study with people with hearing loss. We attempted to mitigate this concern by providing accommodations in the announcement, instructions, and administration of the survey. Plans for on-line administration of the BRFSS may eliminate this barrier in the future.

It is important to note that our survey relied on self-reported hearing loss not verified by audiometric testing. A known limitation of self-report data collected through surveys such as the BRFSS is that the results are not perfectly correlated with audiometric assessments of hearing loss [26]. In terms of the question on hearing ability, the self-report method is likely to have resulted in a mixture of underrepresentation or overrepresentation of actual hearing loss, related to demographic factors such as respondent age, gender, race/ethnicity, and education [19]. For example, among younger adults, individuals tend to over-estimate their self-reported hearing difficulty while older adults tend to under-estimate difficulty.

Yet, there are at least two arguments supporting the use of self-report methods and our result for the purposes of our study. First, based on prior research, interpretation of self-report of hearing ability tends to underestimate overall prevalence among older adults, so use of this data represents a conservative estimate of the population health impacts. Secondly, self-report of hearing disability has been shown to be associated with all major domains of hearing aid outcomes, including: help-seeking, uptake of amplification, use of hearing aids in daily life (treatment adherence), and satisfaction with care [27]. Thus, in terms of estimating population-level needs for audiologic services and other forms of hearing healthcare, self-report of hearing disability is an acceptable starting point.

## Conclusion

This statewide prevalence study of self-reported hearing ability in Arizona and association with health outcomes found that hearing loss was associated with subjective reports of change in cognitive functioning, poorer psychosocial health, and lower self-rated general health among Arizona adults. These findings support the feasibility of assessing self-reported hearing ability on the BRFSS and the usefulness of the results in informing planning processes for programs and services at state and local level.

## Data Availability

The data for this study was generated by the state-based Behavioral Risk Factor Surveillance Survey conducted by the Arizona Department of Health Services. The data and codebook are publically available at: https://azdhs.gov/preparedness/public-health-statistics/behavioral-risk-factor-surveillance/index.php. A limited dataset with geographic identifiers is available on request.

## Abbreviations

BRFSS: Behavioral Risk Factor Surveillance System
CDC: Centers for Disease Control and Prevention
